# Providing eye care services to the indigenous Chepang people of central Nepal

**Published:** 2022-09-20

**Authors:** Manisha Shrestha

**Affiliations:** Pediatric Ophthalmologist: Bharatpur Eye Hospital, Bharatpur, Chitwan, Nepal.

**Community involvement has been critical in eye care delivery to the marginalised Chepang people of Nepal.**

Nepal's national society for comprehensive eye care, Nepal Netra Jyoti Sangh (NNJS), introduced the ‘National Eye Sight Program’ in 2008 with the aim of ‘Reaching the Unreached’ across the country. According to the Nepal Blindness Survey conducted in 1979–80, cataract and childhood blindness were the major eye health challenges in Nepal.^1^

**Figure:**
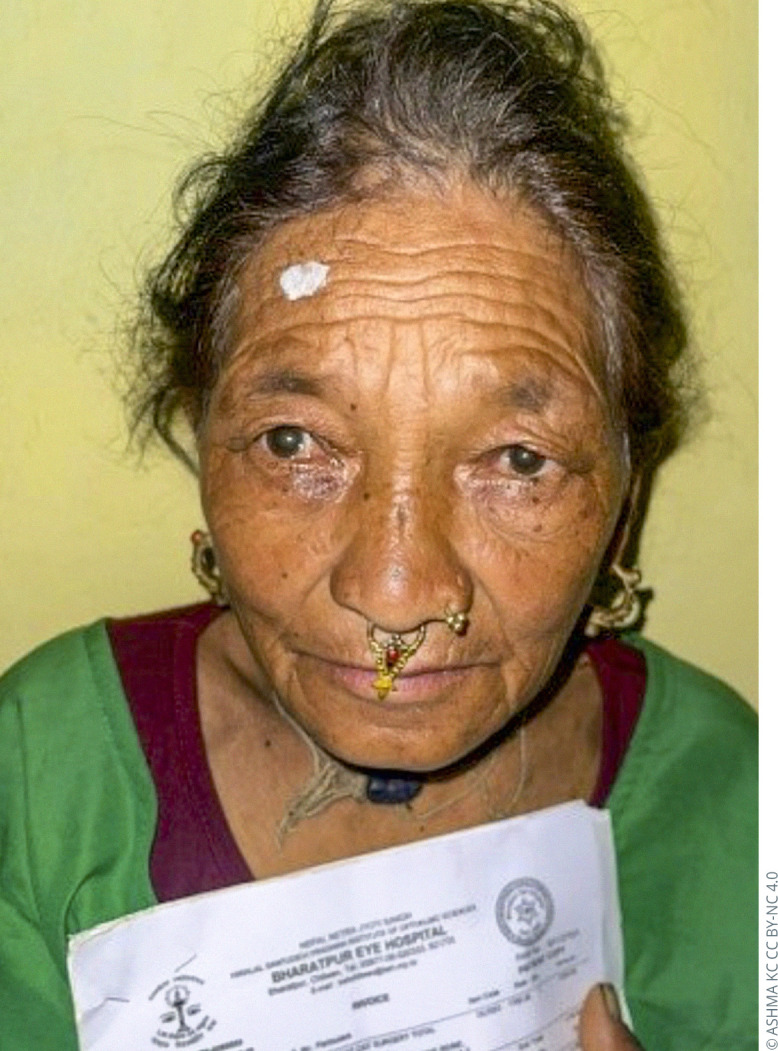
A Chepang woman before surgery at the base hospital. nepal

In order to tackle these challenges and meet the VISION 2020 objectives, NNJS has been conducting eye care outreach activities such as diagnostic, screening, and treatment (DST)camps, surgical eye camps, and school eye health programmes through its eye hospitals and centres across the country, with a focus on reaching marginalised groups.^2^

One of these groups is the nomadic Chepang community, a highly marginalised community of around 70,000 people^3^ who live in the Chitwan, Makwanpur, Gorkha, and Dhading districts in central Nepal. These areas all fall within the catchment area of Bharatpur Eye Hospital.

Eye care services for the Chepang people are provided through mobile diagnostic, screening, and treatment camps. Three or four camps are organised every year, where cataract examinations are carried out, simple cases of conjunctivitis, dry eye, and refractive error are treated, and spectacles are dispensed free of cost to those who need them. People who need surgery are either brought to Bharatpur Eye Hospital or operated on during surgical eye camps, which are organised regularly. A total of 1,143 cataract operations were performed in the year 2020–21.^4^ The programme has a 10-year goal of clearing the treatment backlog of visual impairment among the Chepang community and building community leadership that will improve the uptake of eye health services.

## Community involvement and support

To set up an eye camp, Bharatpur Eye Hospital, in coordination with the district health office, will first establish communication with the head of the village so they can work together to plan the eye camp and recruit volunteers and community motivators, which are both vital to the success of the programme. Volunteers can include younger members of the Chepang community, who have previously worked with immunisation or literacy programmes. Motivators are usually older community members, some of whom have had eye surgery and are satisfied with the treatment. Where possible, school teachers from the neighbourhood and female community health volunteers are mobilised to educated the community about improving their eye health-seeking behaviour. Involving members of the community and showing appreciation for their efforts empowers them and motivates them to further work for their community.

Volunteers are taken to Bharatpur Eye Hospital, where they are taught about the importance of eye health and current measures to end preventable blindness. The volunteers, along with the hospital's field workers, make door-to-door visits in the community, identify those with visual impairment and eye disease, and refer them to the eye camp. The most common eye problems faced by the Chepang are cataract, glaucoma, chronic dacryocystitis, and refractive error.^5^,^6^

## Resource mobilisation

The cost of surgery and other interventions at Bharatpur Eye Hospital is met by donor agencies (in particular, Seva Foundation) who are committed to long-term support. Volunteers use printed information, education, and communication (IEC) material (mainly with figures/illustrations for easy comprehension) to create awareness and disseminate information. The expenses for this and for publicity through FM radio are borne by local non-governmental organisations (NGOs). The cost of food and transportation, basic medicines, and spectacles is covered by NGOs, banks, and philanthropists.

## Challenges and strengths

The challenges to health service delivery are the remote and scattered location of Chepang community members, their lack of formal community leaders, low levels of health awareness, lack of literacy and poor socioeconomic status. Chepang communities are often isolated, and it is usually only the chief male member of the household who ventures outside the community; this tends to keep women, children, and older people away from health facilities and, often, unaware of their existence.

The efforts of the volunteers and motivators from the Chepang community are therefore of the utmost importance in influencing health-seeking behaviour and increasing eye health service uptake among other Chepang people. The motivation of community volunteers, cooperation by community members, the support of NGOs, and the existence of close community clusters all contribute to improved uptake of eye health services. Eventual integration of the programme with the government health service delivery system and financial support from long-term donors also support the programme's long-term sustainability.

**Figure:**
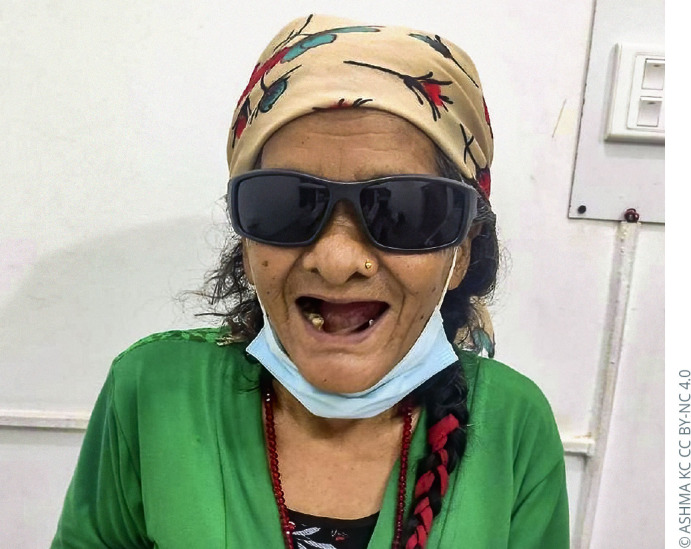
A happy Chepang woman after cataract surgery. nepal
